# Risk Factors Associated with Community-Onset Infections Due to Multidrug-Resistant Organisms

**DOI:** 10.3390/antibiotics14111073

**Published:** 2025-10-25

**Authors:** Rafail Matzaras, Dimitrios Biros, Sissy Foteini Sakkou, Diamantina Lymperatou, Sempastian Filippas-Ntekouan, Anastasia Prokopidou, Revekka Konstantopoulou, Valentini Samanidou, Lazaros Athanasiou, Anastasia Christou, Petros-Spyridonas Adamidis, Amalia Despoina Koutsogianni, George Liamis, Haralampos Milionis, Matilda Florentin, Eirini Christaki

**Affiliations:** 1 1st Department of Internal Medicine & Infectious Diseases Unit, University General Hospital of Ioannina, Faculty of Medicine, School of Health Sciences, University of Ioannina, 45110 Ioannina, Greece; rafail.matz@gmail.com (R.M.); dimitrisbiros@gmail.com (D.B.); andalimperatou@gmail.com (D.L.); sebastienfilippas@gmail.com (S.F.-N.); md06996@uoi.gr (A.P.); revekkakon@gmail.com (R.K.); valentinasmnsmn@gmail.com (V.S.); lazathanasiou@gmail.com (L.A.); hmilioni@uoi.gr (H.M.); 2 2nd Department of Internal Medicine, University General Hospital of Ioannina, Faculty of Medicine, School of Health Sciences, University of Ioannina, 45110 Ioannina, Greece; sissy_sakkou@hotmail.com (S.F.S.); tessie.christou@gmail.com (A.C.); padam7@yahoo.gr (P.-S.A.); amaliadespoina.koutsogianni@gmail.com (A.D.K.); gliamis1@gmail.com (G.L.); mflorentin@uoi.gr (M.F.)

**Keywords:** multidrug-resistant organism, community-onset infection, antimicrobial resistance, Extended-Spectrum Beta-Lactamase (ESBL), antimicrobial stewardship

## Abstract

**Background**: Antimicrobial Resistance (AMR) and the emergence of multidrug-resistant organisms (MDROs) represent major public health threats. Although traditionally linked to hospital-acquired infections (HAIs), MDROs are becoming gradually more prevalent in community-onset infections. **Objectives**: The objective of this study is to identify major risk factors associated with community-onset MDRO infections among patients admitted to the hospital. **Methods**: This is a retrospective study of patients admitted to the Internal Medicine Departments of the University General Hospital of Ioannina from July 2022 to August 2023 and had a microbiologically confirmed infection. Patients with HAIs were excluded. Data were extracted from both electronic and paper-based medical records and included variables such as demographics, baseline comorbidities, previous antibiotic use, previous hospitalizations, the type of MDRO and infection, and clinical outcomes. Statistical analysis included descriptive statistics, univariate analyses, and subsequently multiple binary regression models. Each regression model was adjusted for age and sex. **Results**: Our cohort included 125 participants with a mean age of 77.9 years, with the majority (58.4%) being female. The overall prevalence of MDRO infections was 43.2% (54/125). Notably, the presence of a permanent urinary catheter was associated with a nearly fourfold increase in the risk of community-onset MDRO infections (OR = 3.69; 95% CI: 1.35–10.05; *p* = 0.011), while prior hospitalization (OR = 3.33; 95% CI: 1.48–7.51; *p* = 0.004), the Charlson index score (OR = 3.08; 95% Cl: 1.1–8.68; *p* = 0.033) and previous antibiotic use (OR = 2.18; 95% CI: 0.98–4.84; *p* = 0.057) were also significant potential risk factors. **Conclusions**: The identification of key risk factors associated with community-onset MDRO infections in patients admitted to the hospital can assist clinicians in early stratification and rational selection of initial empirical antimicrobial treatment, support antimicrobial stewardship programs, promote targeted public health interventions, and encourage more judicious antibiotic use.

## 1. Introduction

The emergence of multidrug-resistant organisms (MDROs) has undeniably become a growing public health concern, affecting not only healthcare settings but also the broader community. Although traditionally linked to hospital-acquired infections (HAIs), recent studies have revealed a troubling rise in community-onset infections caused by resistant pathogens—such as methicillin-resistant *Staphylococcus aureus* (MRSA), extended-spectrum *β*-lactamase (ESBL)-producing *Enterobacterales*, and drug-resistant *Streptococcus pneumoniae*. These organisms, which exhibit resistance to multiple classes of antimicrobials, are associated with limited treatment options and increased morbidity and mortality [1,2,3,4].

In community settings, certain populations exhibit risk factors that increase their susceptibility to infections caused by resistant pathogens. Commonly reported factors include advanced age, chronic comorbidities, prior antibiotic exposure, and frequent contact with healthcare environments such as outpatient clinics or long-term care facilities [5,6,7,8]. Older adults, especially those with underlying conditions such as diabetes mellitus (DM), cardiovascular disease, or pulmonary disorders, are particularly vulnerable due to immunosuppression and frequent antimicrobial use [5,6]. The inappropriate or excessive use of antibiotics—especially broad-spectrum agents—has been extensively documented as a key driver of antimicrobial resistance [7]. Moreover, individuals with repeated healthcare exposures, including those undergoing dialysis, living with indwelling devices, or residing in long-term care, are more likely to carry resistant organisms, often asymptomatically [5,8,9,10].

Social determinants of health—such as poor hygiene, overcrowded living conditions, and limited access to healthcare—significantly exacerbate the risk of MDRO transmission within community settings [11,12,13]. Health disparities prevalent in underserved regions may result in delayed diagnoses, suboptimal treatment, and inappropriate antimicrobial use, all of which facilitate the emergence and spread of resistant organisms [12,14].

The objective of this study was to identify the major risk factors associated with community-onset MDRO infections among patients admitted to the hospital. Gaining a better understanding of these potential risk factors is crucial for informing the development of targeted prevention and intervention strategies aimed at reducing the burden of MDRO in the wider community.

## 2. Results

We enrolled a total of 125 participants, with a mean age of 77.9 years (SD: 16.9). Among them, 41.6% were male and 58.4% female. The overall prevalence of MDRO infection was 43.2% (54/125).

Patient demographics, clinical characteristics, microbiologic and laboratory data, and outcomes are shown in Table 1, Table 2 and Table 3. Clinically, participants exhibited a wide range of comorbidities, the most common being hypertension (52.0%), dementia (40.8%), DM (23.2%), congestive heart failure (CHF) (18.4%), stroke (15.2%), asthma or chronic obstructive pulmonary disease (COPD) (15.2%), and CKD (11.2%). Additionally, 21.7% of participants had a permanent urinary catheter, and 15.3% had another type of foreign body. Sepsis and septic shock were observed in 44.6% and 24.0% of the participants, respectively. The overall in-hospital mortality rate was 24.8%, and the admission rate to the ICU was 0.8%. The mean duration of antibiotic therapy was 11.3 days (SD: 7.3), while the average length of hospital stay was 10.9 days (SD: 8.6).

Among microbiological isolates obtained from clinical specimens, 22.4% were positive for ESBL-producing organisms, and 2.4% for MRSA, while no Vancomycin Resistant *Enterococci* (VRE) isolates were detected. Furthermore, 7.3% of participants were infected with carbapenem-resistant organisms, defined in this study as *Enterobacterales*, *Acinetobacter baumannii*, and *Pseudomonas aeruginosa*, but not *Stenotrophomonas maltophilia*, while colistin-resistant strains were identified in 4.0% of participants, referring exclusively to *Enterobacterales*, *Acinetobacter baumannii*, and *Pseudomonas aeruginosa*.

Among participants with recorded laboratory values, the median C-reactive protein (CRP) level was 106.4 mg/L (SD: 93.8), the mean procalcitonin (PCT) level was 7.1 ng/mL (SD: 20.9), and the mean white blood cell count was 12,974 cells/μL (SD: 6490). 

**Figure 1:**
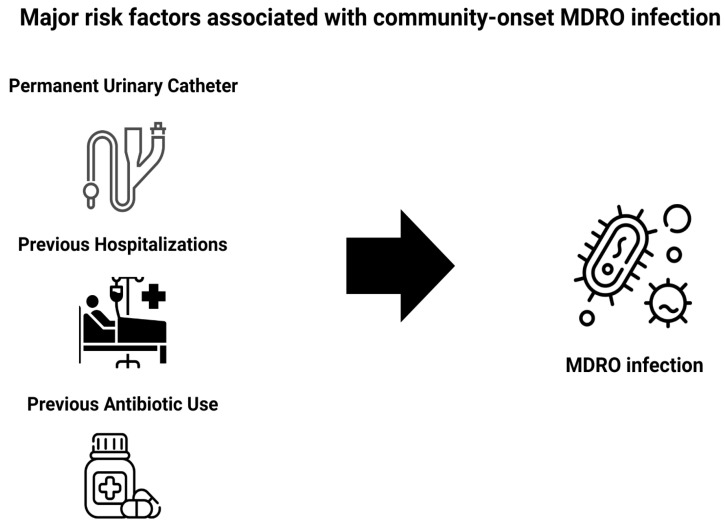
Risk factors associated with a community-onset MDRO infection, identified by a logistic regression.

To identify independent predictors associated with MDRO infection, we conducted a series of logistic regression models, each adjusted for age and sex (Table 4). Prior hospitalization within the last 12 months was associated with a significantly higher risk of MDRO infection (OR = 3.33; 95% Cl: 1.48–7.51; *p* = 0.004) Residence in a healthcare facility, as opposed to living at home, and previous antibiotic use were also significant potential risk factors (OR = 1.87; 95% Cl: 0.76–4.59; *p* = 0.171 and OR = 2.18; 95% CI: 0.98–4.84; *p* = 0.057, respectively). Notably, the presence of a permanent urinary catheter was associated with a nearly fourfold increase in MDRO infection risk (OR = 3.69; 95% CI: 1.35–10.05; *p* = 0.011). Other variables, including chronic liver disease, DM, CKD, and hypertension, were not significantly associated with MDRO infection in multivariate analyses except for the Charlson index score (OR = 3.08; 95% CI: 1.1–8.68; *p* = 0.033). The major risk factors associated with community-onset MDRO infection are schematically presented in Figure 1.

## 3. Discussion

In this retrospective case–control study of 125 patients with microbiologically confirmed community-onset infections, nearly half of them (43.2%) were found to have an infection caused by an MDRO. Our analysis identified recent hospitalization in the last year, the presence of a permanent urinary catheter, the Charlson comorbidity index, and previous antibiotic use in the last three months as the strongest independent risk factors associated with community-onset MDRO infections. In contrast, common comorbidities such as DM, CKD, and hypertension were not significantly associated.

The recognition of risk factors associated with community-onset MDRO infections is critical for developing targeted prevention strategies and guiding empiric antimicrobial therapy. These infections are shaped by a complex interplay of healthcare exposure, sociodemographic variables, environmental factors, and antibiotic use practices in outpatient settings. Identifying key determinants not only informs clinical decision-making but also supports broader public health interventions aimed at limiting the spread of resistance within the community.

While the frequency, risk factors, and outcomes of healthcare-associated MDRO infections have been well established [6,7,8,9,10], emerging evidence highlights the growing presence of MDROs in community-acquired infections. Residence in long-term care or rehabilitation facilities has been consistently associated with MDRO colonization or infection [15,16,17]; however, recent data indicating that individuals residing in community may also be at risk of community-onset MDRO infections make it critical to identify those at higher risk, given the association of MDROs with worse outcomes [15]. Notably, most previous studies have focused on specific MDROs or isolated infection types, like bacteremia or pneumonia. In contrast, our study encompassed a wide range of clinically established infections, offering a more comprehensive assessment of resistance patterns in real-world community settings.

Multiple factors contribute to the acquisition of MDRO infections, including both clinical characteristics and healthcare exposures. Among these, prior antibiotic use stands out as one of the most consistently reported potential risk factors [16,17]. Broad-spectrum antibiotic use, especially when recent, can disrupt the host microbiota, facilitating the selection and proliferation of resistant organisms [18]. Previous hospitalization, particularly in high-risk settings such as ICUs, similarly increases exposure to MDROs commonly found in healthcare environments [19,20].

Our findings, thus, align with established evidence that recent antibiotic use and hospitalization within the past year have been independently associated with an increased risk of community-onset MDRO infection [17,21,22]. These results highlight the growing overlap between community and healthcare-associated risk factors, underscoring the need to reassess what constitutes a “community-onset” infection in current clinical practice.

Invasive devices such as urinary catheters and foreign bodies are also well-documented potential risk factors serving as entry points and surfaces for biofilm formation and microbial colonization [21,22,23,24,25]. Furthermore, previous infection or colonization with an MDRO can predispose individuals to subsequent infections with similar resistant organisms [20]. In our study, permanent urinary catheters were one of the strongest predictors associated with community-onset MDRO infections. While comorbidities such as DM, CKD, and malignancy have been previously linked to increased infection susceptibility [18], we did not observe a statistically significant association in multivariate analysis. One possible explanation for this lack of association could be the relatively small sample size, since a small number of patients with these comorbidities reduces the ability to detect a statistically significant correlation. Also, single comorbidities may not be strong predictors for colonization with MDROs. However, a Charlson Comorbidity Index >4 was suggestive of a higher MDRO risk (OR = 3.08, *p* = 0.033), pointing to a potential role for cumulative disease burden [26,27].

In our study, the most frequently isolated pathogens were ESBL-producing *Enterobacterales.* Importantly, these organisms are no longer confined to HAIs [28]. Community-onset ESBL infections are rising worldwide, with prevalence varying by geography and patient population. Globally, reported rates vary widely, ranging from <2% in Norway [29] to as high as 74% in Iraq [30]. In a multicenter prospective study conducted in the United States between 2009 and 2010, 4% of community-onset *E. coli* isolates were identified as ESBL-producing, most frequently associated with UTIs [31]. In China, ESBL-producing *Klebsiella pneumoniae* accounted for 31.8% of community-onset infections, a rate comparable to that observed in hospital-acquired cases [32]. Moreover, a retrospective study documented a sharp increase in ESBL-producing *E. coli* causing community-onset bacteremia, from 3.6% in 2006 to 14.3% in 2011 [18].

Correspondingly, a meta-analysis of 34 studies including 4528 isolates collected between 2007 and 2023 reported an overall prevalence of ESBL-producing *Enterobacterales* in Egypt of 65% in community-acquired infections versus 62% in nosocomial infections (*p* = 0.68), highlighting the extensive circulation of these strains outside hospital settings [33]. Similarly, in Qatar, a retrospective study of adult patients with ESBL-UTIs found that 25.2% were community-onset [34], and in Taiwan, 13.5% of 393 patients with community-onset UTIs were caused by ESBL-producing microorganisms, with *E. coli* being the most commonly isolated species [35]. Also, at the same time, accumulating evidence highlights a marked increase in community carriage of ESBL-producing organisms. A meta-analysis reported that community carriage of ESBL-producing *E. coli* rose dramatically from 2.6% (95% CI: 1.2–4.0%) in 2001–2005 to 26.4% (95% CI: 17.0–35.9%) in 2016–2020, reflecting an approximately tenfold increase [36].

Regarding risk factors associated with ESBL infections, colonization with ESBL-producing *Enterobacterales*, prior empiric antibiotic use, recent hospitalization, and urinary catheterization are considered the most relevant [37,38,39,40]. A systematic review and meta-analysis of 22 studies, including 10,570 long-term care facility residents, estimated that residence in such facilities is itself significantly associated with ESBL colonization, with a pooled prevalence of 15.8% (95% CI: 0.04–31.53, varying by region). Empiric antibiotic use, recent hospitalization, and presence of urinary catheter use were also identified as important risk factors [37]. Another systematic review of 16 observational studies (N = 12,138 patients) further confirmed that prior antibiotic use (OR range: 2.2–21.4), previous hospitalization (OR: 1.7–3.9), and history of urinary tract infection (OR: 1.3–3.8) represent strong predictors associated with ESBL infections [40].

In addition, a study from New Zealand identified several independent potential risk factors for bacteremias caused by ESBL-producing *Enterobacterales*: known colonization with an ESBL-producing organism (OR: 46.2, 95% CI: 3.45–619), exposure within the preceding year to first-generation cephalosporins (OR: 12.3, 95% CI: 1.01–148) or fluoroquinolones (OR: 6.56, 95% CI: 1.79–24), and prolonged cumulative hospital stay (OR: 1.033, 95% CI: 1.001–1.066 per inpatient day) [38].

Several important limitations should be acknowledged in this study. The retrospective design introduces potential selection bias, as the data extracted were based on existing records. Furthermore, since the study relied on pre-existing medical records, information may be incomplete or absent. Retrospective studies also limit the ability to establish causal relationships due to the inability to control for unmeasured confounders. The single-center setting restricts external validity and generalizability, and the relatively small sample size reduces statistical power. Moreover, despite adjustment for age and sex, residual confounding may persist due to unaccounted factors. The combination of these constraints necessitates cautious interpretation of results and highlights the need for validation in larger, multicenter, prospective studies before drawing definitive conclusions. As emphasized in current guidelines, antimicrobial stewardship programs should be guided by local epidemiological data to design targeted and effective interventions [41]. Our study reflects a real-world cohort of patients with community-onset infections, in whom risk factors for MDROs were systematically evaluated. In clinical practice, it is often assumed that patients with traditional risk factors harbor MDROs, leading to the empirical use of broad-spectrum antibiotics-even in cases of non-severe infections. It is, therefore, essential to define certain, evidence-based risk factors for community-onset MDRO infections in order to optimize empirical antimicrobial therapy, particularly in countries with a high prevalence of antimicrobial resistance in hospital settings.

## 4. Materials and Methods

### 4.1. Study Design

We conducted a case–control study to identify the risk factors associated with community-onset infections caused by MDROs among patients hospitalized at a tertiary university hospital in Greece.

### 4.2. Study Population

The study included adult patients (≥18 years) who were admitted to the 1st and 2nd Department of Internal Medicine of the University General Hospital of Ioannina between July 2022 and August 2023 with microbiologically confirmed community-onset infections.

Patients with HAIs were excluded. Because residents of long-term care facilities may develop infections caused by resistant or wild-type pathogens, these patients were not excluded solely on the basis of residence. Additional exclusion criteria included: (1) cases of colonization without clinical infection (i.e., without local or systemic signs of infection and without abnormal values of inflammatory markers), (2) potentially contaminated specimens—such as coagulase-negative staphylococci isolated from a single blood culture—and (3) asymptomatic bacteriuria without systemic signs or symptoms.

Cases were defined as adult patients with microbiologically confirmed MDRO infections diagnosed within 48 h of admission. MDRO classification and infection type criteria were based on the Centers for Disease Control and Prevention (CDC) and European Centre for Disease Prevention and Control (ECDC) definitions. MDR is defined as non-susceptibility to at least one agent in three or more antimicrobial categories [42]. Eligible infection types included: Bloodstream infections (BSIs), urinary tract infections (UTIs), skin and soft tissue infections (SSTIs), gastrointestinal infections (GIIs), and lower respiratory tract infections (RTIs).

Controls were patients aged ≥ 18 years who were hospitalized during the same period and diagnosed with infections caused by non-MDRO pathogens. For each case, three controls were matched based on age and hospital unit of admission. The same infection types and diagnostic criteria were applied to both groups (discussed above).

### 4.3. Data Collection

Data were extracted from both electronic and paper-based medical records. Variables collected included: demographics (age, sex, and residence in chronic care facility), comorbidities (e.g., diabetes mellitus, hypertension, chronic liver disease, congestive heart failure, coronary artery disease, asthma, chronic obstructive pulmonary disease, stroke, dementia, connective tissue disease, peripheral artery disease, chronic ulcers or other skin lesions, active malignancy, immunodeficiency), history of MDRO infection or known colonization within the past 12 months, antibiotic use within the previous 3 months, and hospitalizations within the past 12 months, invasive procedures within 1 month prior to admission, presence of indwelling devices (central venous catheter, urinary catheter, PEG tube) or implanted foreign bodies (e.g., pacemakers, vascular grafts, joint prostheses), history of chronic kidney disease (CKD) or renal replacement therapy. Microbiological data were collected, including pathogen identification, antimicrobial resistance profiles, type of infection, antibiotics administered, and treatment duration. Last, severity of illness, Sequential Organ Failure Assessment [SOFA] score, length of hospital stay, Intensive Care Unit (ICU) admission, in-hospital mortality, and clinical outcome were recorded.

### 4.4. Data Management

Data collection followed a standardized protocol. User manuals were developed to guide consistent data entry, and training meetings were held with principal investigators prior to study initiation to ensure uniform data recording across all participating personnel. Laboratory and microbiological data were obtained from the Microbiology Department of the University General Hospital of Ioannina, following their validated methodologies and established good laboratory practices. All data entries were cross-checked for accuracy. Anonymization procedures were applied prior to analysis to ensure the confidentiality of patient information.

### 4.5. Ethical Considerations

The study protocol was reviewed and approved by the Institutional Review Board of the University General Hospital of Ioannina. All patient data were anonymized, and access was restricted to authorized members of the research team in accordance with ethical research standards and the General Data Protection Regulation (GDPR). The requirement for written informed consent was waived due to the retrospective design of the study.

### 4.6. Statistical Analysis

Demographic and clinical characteristics of the study population were summarized using descriptive statistics. Categorical variables were reported as frequencies and percentages, while continuous variables were presented as means with standard deviations (SD) for normally distributed data. Univariate analyses were initially conducted to assess the distribution and frequency of individual variables. Subsequently, multiple binary logistic regression models were performed to evaluate the association between community-onset MDRO infections and selected independent variables, including: residency type, prior antibiotic use, previous hospitalizations, urinary catheterization, presence of gastrostomy, end-stage renal disease (ESRD), history of trauma, presence of foreign bodies, and relevant comorbidities. Each regression model was adjusted for patient age and sex. The results were reported as odds ratios (ORs) with 95% confidence intervals (CIs) to quantify the strength of associations. A *p*-value of <0.05 was considered statistically significant. Analyses were performed using IBM SPSS Statistics version 26. Participants with incomplete data (system-missing values) were excluded from each respective analysis. Frequencies/Percentages and Means were calculated based on the valid (non-missing) values for each variable. The proportion of excluded cases per variable was minimal.

## 5. Conclusions

We identified key risk factors associated with community-onset infections caused by MDROs in patients requiring hospitalization. Prior antibiotic use, the presence of a permanent urinary catheter, and hospitalization within the previous twelve months were significantly linked to an increased risk of MDRO infections. By highlighting these factors, our findings aim to assist clinicians in early risk stratification and support antimicrobial stewardship programs in promoting targeted infectious disease management and more judicious antibiotic use.

## Figures and Tables

**Table 1:** Patient demographics and clinical characteristics.

Variable	Mean (SD) or N (%)
**Demographics**	
Age (years)	77.9 (N = 125, SD: 16.9)
Sex	
Female	73/125 (58.4%)
Male	52/125 (41.6%)
Residence before hospital admission	
Home	93/124 (75%) *
Healthcare facility	31/124 (25%) *
**Clinical Characteristics**	
Previous MDRO infections within 12 months	9/119 (7.6%) *
Known colonization with an MDRO	7/117 (6%) *
Previous antibiotic use within 3 months	46/123 (37.4%) *
Previous hospitalizations within 12 months	54/118 (45.8%) *
History of invasive procedure within 1 month (surgery or biopsy)	3/119 (2.5%) *
History of non-invasive procedure within 1 month (colonoscopy or cystoscopy)	8/118 (6.8%) *
CVC	1/119 (0.8%) *
Permanent urinary catheter	26/120 (21.7%) *
Presence of foreign bodies (pacemaker, graft, or joint prostheses)	18/118 (15.3%) *
History of CKD into renal replacement	1/119 (0.8%) *
History of IV infusion therapy at home	5/119 (4.2%) *
Presence of gastrostomy	2/120 (1.7%) *

CKD: chronic kidney disease, CVC: central venous catheter, IV: intravenous, MDRO: multi-drug resistant organism. * Frequencies/Percentages and Means were calculated based on the valid (non-missing) values for each variable. The proportion of missing values per variable was minimal.

**Table 2:** Patient’s Comorbidities.

Variable	N (%)
Dementia	51/125 (40.8%)
Hypertension	65/125 (52%)
DM	29/125 (23.2%)
CHF	23/125 (18.4%)
Stroke	19/125 (15.2%)
CAD	8/124 (6.5%) *
PAD	4/125 (3.2%)
CKD	14/125 (11.2%)
Asthma or COPD	19/125 (15.2%)
Immunosuppression	15/125 (12%)
Active malignancy	9/125 (7.2%)
Connective tissue disease	6/125 (4.8%)
Chronic Liver Disease	5/125 (4%)
History of transplantation	1/125 (0.8%)
Trauma	2/120 (1.7%) *
Chronic ulcers or other skin lesions (i.e., decubitus ulcer, diabetic foot, postoperative wound)	10/125 (8%)

CAD: coronary artery disease, CHF: congestive heart failure, CKD: chronic kidney disease, COPD: chronic obstructive pulmonary disease, DM: diabetes mellitus, PAD: peripheral artery disease, * Frequencies/Percentages and Means were calculated based on the valid (non-missing) values for each variable. The proportion of missing values per variable was minimal.

**Table 3:** Microbiological data, Inflammatory markers, and Outcomes.

Variable	Mean (SD) or N (%)
Duration of antibiotics	11.3 days (N = 119, SD: 7.3) *
MDRO	39/125 (31.7%)
ESBL	28/125 (22.4%)
MRSA	3/125 (2.4%)
VRE	0/125 (0%)
VRSA	0/125 (0%)
VISA	1/125 (0.8%)
Resistance to carbapenems	9/124 (7.3%) *
Resistance to colistin	5/124 (4%) *
Length of hospital stay	10.9 (N = 120, SD: 8.6) *
Sepsis	54/121 (44.6%) *
Septic shock	29/121 (24%) *
ICU admission	1/120 (0.8%) *
Death	30/121 (24.8%) *
WBCs (cells/μL)	12,974 (N = 125, SD: 6490)
PCT (ng/mL)	7.1 (N = 54, SD: 20.9) *
CRP (mg/L)	106.4 (N = 124, SD: 93.8) *
Lactic Acid (mmol/L)	1.85 (N = 119, SD: 1.4) *

CRP: C-Reactive Protein, ESBL: Extended-spectrum *β*-lactamase, ICU: Intensive care unit, MDRO: Multi-drug resistant organism, MRSA: Methicillin-resistant Staphylococcus aureus, PCT: Procalcitonin, VRE: Vancomycin-resistant enterococci, VRSA: Vancomycin-resistant Staphylococcus aureus, VISA: Vancomycin-intermediate Staphylococcus aureus, WBCs: White Blood Cells, * Frequencies/Percentages and Means were calculated based on the valid (non-missing) values for each variable. The proportion of missing values per variable was minimal.

**Table 4:** Binary logistic regression analysis of factors associated with an MDRO infection.

Variable	OR	*p*-Value	95% CI
Healthcare facility residence (vs home)	1.87	0.171	0.76–4.59
Recent antibiotic use within 3 months	2.18	0.057	0.98–4.84
Previous hospitalizations within 12 months	3.33	0.004	1.48–7.51
Permanent urinary catheter	3.69	0.011	1.35–10.05
Presence of foreign body (pacemaker, graft, or joint prostheses)	1.59	0.383	0.56–4.52
Charlson index score	3.08	0.033	1.1–8.68
DM	1.67	0.263	0.68–4.07
Hypertension	1.27	0.530	0.60–2.72
Chronic liver disease	0.84	0.859	0.13–5.63
CKD	0.81	0.737	0.24–2.78
CHF	0.81	0.684	0.30–2.19
CAD	1.18	0.833	0.26–5.24
COPD	1.83	0.258	0.64–5.21
Stroke	2.06	0.182	0.71–5.94
Dementia	2.07	0.065	0.96–4.5
CTD	2.03	0.440	0.34–12.17
PAD	0.309	0.323	0.03–3.17
Chronic ulcers or other skin lesions (i.e., decubitus ulcer, diabetic foot)	1.89	0.365	0.48–7.47
Active malignancy	1.4	0.636	0.35–5.69
Immunodeficiency	0.455	0.192	0.14–1.48

CAD: Coronary Artery Disease, CHF: Congestive Heart Failure, CI: Confidence Interval, CKD: Chronic Kidney Disease, COPD: Chronic Obstructive Pulmonary Disease, CTD: Connective Tissue Disease, DM: Diabetes Mellitus, OR: Odds Ratio, PAD: Peripheral Artery Disease.

## Data Availability

The original contributions presented in this study are included in the article. Further inquiries can be directed to the corresponding author.

## Abbreviations

The following abbreviations are used in this manuscript:

AMR	Antimicrobial Resistance
BSI	Bloodstream Infection
CAD	Coronary Artery Disease
CDC	Centers for Disease Control and Prevention
CHF	Congestive Heart Failure
CI	Confidence Interval
CKD	Chronic Kidney Disease
COPD	Chronic Obstructive Pulmonary Disease
CRP	C-Reactive Protein
CTD	Connective Tissue Disease
CVC	Central Venous Catheter
DM	Diabetes Mellitus
ECDC	European Centre for Disease Control and Prevention
ESBL	Extended-Spectrum *β*-lactamase
ESRD	End-Stage Renal Disease
MDRO	Multidrug-Resistant Organism
GDPR	General Data Protection Regulation
GII	Gastrointestinal Infection
HAI	Hospital-Acquired Infection
ICU	Intensive Care Unit
MRSA	methicillin-resistant *Staphylococcus aureus*
OR	Odds Ratio
PAD	Peripheral Artery Disease
PCT	Procalcitonin
RTI	Respiratory Tract Infection
SD	Standard Deviation
SOFA	Sequential Organ Failure Assessment
SSTI	Skin and Soft Tissue Infection
UTI	Urinary Tract Infection
VISA	Vancomycin Intermediate *Staphylococcus aureus*
VRE	Vancomycin Resistant *Enterococci*
VRSA	Vancomycin Resistant *Staphylococcus aureus*
WBC	White Blood Cell
